# Does knowledge and concern regarding food supplement safety affect the behavioral intention of consumers? An experimental study on the theory of reasoned action

**DOI:** 10.3389/fnut.2023.1305964

**Published:** 2024-01-08

**Authors:** Talha Bayır, Selim Çam, Murat Fatih Tuna

**Affiliations:** ^1^Sirnak University, Sirnak, Türkiye; ^2^Cumhuriyet University, Sivas, Türkiye

**Keywords:** health consciousness, food safety knowledge, food safety concern, theory of reasoned action, food supplement, consumer behavior

## Abstract

In recent years, health crises have led consumers to make more frequent purchases of food supplements. The global food supplement market, which reached $61.20 billion in 2020, is estimated to reach $163.12 billion by 2022 and $350.96 billion by 2032. However, many consumers still have concerns about the safety of food supplements. Within the scope of the research, firstly, the health consciousness (HC) level of food supplement consumers was determined. Secondly, food safety knowledge (FSK) and food safety concerns (FSCs) were measured. Thirdly, consumers’ attitudes (ATUs), subjective norms (SNs), and behavioral intentions (BIs) toward food supplements were determined within the scope of the theory of reasoned action. The study used a convenient sampling, and 327 participants were included in the sample population. The data for the analysis was collected using the online survey method in the third quarter of 2023. The relationships between hypothesized items in the structural model were tested using the Smart-PLS. The validity and reliability of the measurement model were evaluated at the start of the structural equation modelling approach using confirmatory factor analysis (CFA). Regression analyses were performed in the structural model phase to evaluate overall fit and suggested relationships by way of the Smart-PLS. In light of the findings, it was determined that the interaction between HC and ATU was mediated by FSK, and the interaction between HC and the SN was mediated by FSK. Consequently, this research presents a variety of theoretical and practical implications to give clues for consumers’ health regarding food supplement consumption.

## Introduction

Food is a major social issue closely related to people’s lives and is becoming increasingly critical due to this relevance (1). One of the main goals in solving this problem has been shown by the United Nations ensuring food security (2). As certain factors, such as globalization, urbanization, increasing disposable income, and purchasing preferences, continue to change dietary habits worldwide, food security (FS) concerns have increased (3). The statistics on food safety are staggering. By the World Health Organization (4), approximately one in ten people worldwide suffer from an illness due to the consumption of contaminated food, and 420 thousand dies as a result annually. According to the same statistics, children under five carry 40% of the foodborne disease burden, with 125 thousand deaths annually (4). It is also known that food safety problems create a budget burden. While foodborne illness costs the American food service industry $55.5 billion annually, each food safety outbreak costs the business between $6,330 and $2.1 million, depending on the company’s size and how widespread the outbreak is (5). These figures make the perception of food safety important for the public and for consumers who are part of the public. According to Knight and Warland (6), although farmers, businesses and government agencies take steps to ensure a safe food supply, food safety ultimately depends on public perception. On the other hand, consumers’ ecological and genetic concerns about food safety led them to be informed about food (7). Therefore, it is critical to investigate the impact of consumers’ knowledge and concerns about food safety on their behavioral intentions (BIs). Indeed, Liguori et al. (3) state that consumers’ concerns about food safety affect their food consumption behavior and dietetic attitudes.

Food safety was first used to describe whether a country had access to sufficient food to meet its nutritional energy requirements (8). Over time, the definition of the concept has been expanded to include the handling, processing, preparation, and storage of food in a way that helps prevent foodborne diseases (9). Although there is a theoretical study on food safety (10), several studies also address the issue from a consumer perspective. A number of these studies aim to measure the knowledge and practicality of consumers regarding food safety. At this point, Unusan (11) and Kennedy et al. (12) investigated the food safety perceptions of consumers who prepare food for their households and how they apply it in practice. Medeiros et al. (13) even developed a knowledge and attitude scale that can be used in consumers’ food safety education. Empirical studies aim to increase consumers’ knowledge and practice through education in adults (14, 15) and children (16, 17). Several studies in the literature focus on consumers’ perceptions of food safety in terms of the service sector. In one of these studies, Liu and Lee (18) reduced restaurant consumers’ perceptions of food safety into functional, mechanical and human perspectives. In another study, Seaman, and Eves (19) investigated the role of hygiene in managing food safety in the service sector.

Despite the growing popularity of food safety as a topic of study, there are relatively few studies on the BIs of dietary supplement consumers (20, 21). To date, academics have used the protection motivation theory (PMT) (22, 23), the health belief model (HBF) (24), the value-attitude-behavior (VAB) (25) and the theory of planned behavior (TPB) (9, 26) as well as the theory of reasoned action (TRA) (22, 27) to investigate consumers’ perceptions and attitudes towards food safety.

The TRA, one of these theories, is a theory developed by Fishbein & Ajzen (28) that attempts to explain BI with ATUs and subjective norms (SNs). It can be seen that the studies that want to benefit from the explanatory power of the TRA within the scope of food safety have focused on food safety and risk communication (22), food safety intention (29) and working habits of food industry employees (30), attitudes and beliefs of consumers with regard to food hygiene (27), the creation of segmentation strategies related to genetically modified foods (31), halal food purchasing behavior (32), organic food purchasing behavior (33) and whether such behavior differs according to gender (34). In addition, the mentioned studies investigate the BIs of consumers, food sector employees, managers and consumers towards food.

Another theory, the TPB, emerged by adding another intention determinant, called perceived behavioral control, to the TRA (35). Accordingly, the studies in which it is used within the scope of food safety have investigated the issue based on farmers (36, 37), food business employees (38, 39), restaurant managers (40), and consumers (41, 42). It can also be seen that studies using the TPB based on consumers have addressed food safety in the hygienic dimension, which is a sub-dimension related to food safety (42, 43), in a limited period such as the pandemic (9, 44), and in the direction of informing specific consumer groups such as low-income families (45). In addition, studies using the theory have addressed safe food consumption (43), improving food safety measures (46), healthy eating (47), healthy and sustainable food purchasing behavior (48), organic food consumption (49, 50), the role of trust in organic food consumption (51), BI regarding food safety (52), intention to purchase halal food (53), food supplement purchasing behavior (20, 21, 54), and the effects of food safety on BI (52).

Although there are studies that address food safety and food supplement issues together (55, 56), or even aim to explain consumer behavior by using more than one theory together [for example, PMT + TPB (22); VAB + TPB (57); TRA + TPB (58)], there is no study that addresses the mentioned issues together, includes food safety knowledge (FSK) and concern in the model and aims to explain consumers’ BIs with the TRA and TPB. In addition, no study has tried to explain FSK and food safety concern (FSC) with the TRA.

FSC and FSK are topics addressed with health consciousness (HC) in the literature. In a study using HC and FSC as variables, Michaelidou and Hassan (59) examined the effect of ethical identity on the attitude of consuming organic products. On the other hand, Hsu et al. (60) investigated the effect of FSC and HC on intention to purchase organic products. Quick et al. (61) investigated the effect of FSK on BI of secondary school students using the HBF and the TRA. Khayyam et al. (9) examined the effect of food safety and HC on consumption behavior intention in the context of the TPB. The literature also rarely shows that FSCs affect HC. Su et al. (62) suggested that FSC is one of the factors affecting HC. However, none of the mentioned studies established a model to predict the relationship between FSC and HC. On the other hand, Nagaraj (63) revealed a relationship between FSC and HC and that this relationship affects the intention to purchase organic products through consumer attitude.

FSK, another dimension addressed with HC, is a less studied topic than FSC. Shafieizadeh et al. (64) stated that adopting food safety information is influenced by perceived information quality and perceived information reliability and that HC and FSK moderate this effect. Although there have been studies that overlap the perception of food safety with the BI of healthy food knowledge (65), to the best of our knowledge, there is no study that uses the TRA theory to explain the moderating effect of consumers’ knowledge and concerns regarding food safety on their BIs. Moreover, since studies on food safety have focused on several different aspects, it is evident that there is a lack of studies that precisely portray the knowledge and concerns of consumers who purchase food supplements.

In addressing the gaps, this study develops and examines a holistic model. The study will provide an understanding of the role of FSK and concerns in forming BIs regarding food supplements. In this way, it will make a tangible contribution to the widespread literature. In addition, the study’s results will contribute to businesses in the food supplement market, which is a growing market, helping them to understand their customers better. With this study, food supplement providers are thought to take the following steps to provide the necessary information by addressing consumers’ concerns.

## Literature review and hypothesis development

### Food supplement

Technology has made it possible to produce standard nutrients in powders and tablets and thereby easily integrate them into a regular diet (66). The gradual growth of the sector has led to the common name of food supplements. The European Food Safety Authority (EFSA) (67) defines food supplements as follows: “Food supplements are concentrated sources of nutrients (i.e., minerals and vitamins) or other substances with a nutritional or physiological effect that are marketed in ‘dose’ form (e.g., pills, tablets, capsules, liquids in measured doses).” The current understanding of health requires patients to take a more active role in their own health care and, for many people, being ‘healthy’ means taking dietary supplements (68). Food supplements include minerals, vitamins, favoured carbohydrates, pre-probiotics, essential fatty acids, amino acids, fiber-containing supplements, various plants, and extracts from these plants. It is necessary to ensure that food supplements are safe before they are marketed, and the product label should reflect accurate information that is not misleading (69). Apart from their basic properties, according to Stoś et al. (70), food supplements are seen to mitigate the impact of unhealthy diets for young consumers, and to maintain good health for adults. Food supplements are a growing industry, and the statistics are impressive. The size of the global dietary supplement market was $163,986 million in 2022 and is projected to increase by 9% annually by 2030 (71).

### Health consciousness

HC refers to the state of readiness to undertake health actions (72). Previous studies have shown that health is perceived as an individual investment and a determinant of consumers’ purchase intentions (73). Health-conscious consumers are aware of and concerned about their own well-being and are motivated to improve and/or maintain their health and quality of life; they also prevent diseases by engaging in health-conscious behavior (59). In addition, consumers with this consciousness show a BI to consume healthy products (9). Previous studies on HC are generally associated with the consumption of organic products (60, 63) and healthy food (74). In addition, food supplements are one of the functional food’s individuals use, and their tendency to consume these supplements is influenced by HC (75). HC is also an essential determinant of attitudes towards food supplements and price perception (68). In addition, it has also been reported in the literature that the HC of restaurant consumers affects their BIs and purchase decisions (76), which is in line with the findings of Nagaraj (63). Again, Nagaraj (63) emphasized that there is a causality between HC and FSC in his study. However, unlike the current study, he focused on the mediating role of FSC. The following hypotheses were developed in line with the literature review:

*H1*: The health consciousness scale positively affects the food safety knowledge scale.

*H2*: The health consciousness scale positively affects the food safety concern scale.

### Food safety knowledge

Increased awareness of food safety has led consumers to need more information on the chemical content of foods (77). When a person knows that a food is safe, he/she has more control over purchasing the food (78). Although this knowledge differs according to demographic variables (79), it seems to moderate the intention to buy organic food (80). On the other hand, it is known that consumer knowledge about safe and healthy products creates a preference and awareness for these products (81). In support of this, Kashif et al. (82) state that consumers’ knowledge has a regulatory role in buying healthy and organic products. Therefore, it can be expected that as consumers gain knowledge about food safety, they will develop attitudes and intentions towards purchasing, and this assertion has a counterpart in the literature (64). Accordingly, for restaurants to create trust in their establishments and purchase intentions towards their products, consumers need to accept that the food served in the restaurant is safe (64). At this point, Chan et al. (83) link it to the development of FSK of those students who will be trained to work in food production enterprises. In another study in which the food safety of university students was investigated, Sanlier and Konaklioglu (84) found that FSK also differed according to gender and the institution where students received their education. The following hypotheses were developed in line with the literature research:

*H3*: The food safety knowledge scale positively affects attitude toward using the scale.

*H4*: The food safety knowledge scale positively affects the subjective norm scale.

### Food safety concern

FSCs represent consumers’ concerns regarding residue in food from chemical sprays, fertilizers, artificial additives, and preservatives often linked to farming methods (85). In parallel, according to the Special Eurobarometer Wave EB97.2 report prepared by EFSA, the top three most frequently selected concerns of consumers are (i) pesticide residues in food (40%); (ii) antibiotics and hormones in meat (39%); and (iii) preservatives used in food and/or beverages (36%) (86). Hsu et al. (60) found that FSCs contribute to developing attitudes towards organic products and purchase intention. This result makes FSC one of the main variables investigated in studies investigating food safety in different dimensions (3, 6, 14, 36). In the literature review on the nature of FSC in terms of food supplements, it can be seen that studies mainly focus on the compliance of foods with food safety standards in terms of the substances they contain (55) and the quality of the substances contained in foods (87). This situation is associated with the low acquisition costs of food supplements (88) and the content prone to falsification (89). Therefore, it can be said that FSCs affect the shaping of individuals’ HC. Michaelidou and Hassan (59) show that FSC cannot directly form food purchase intention, but that it can do so through organic product purchase attitudes. On the other hand, no relationship was reported between HC, another variable used in the study, and FSC, a similar approach followed by Hsu et al. (60) and Bhutto et al. (73). In addition, Nagaraj (63) hypothesizes that there is a relationship between HC and food safety in the direction of purchasing organic food. However, he could not reveal the existence of the relationship because he acted on the assumption that there was a direct relationship. In line with the literature research, the following hypotheses were developed:

*H5*: The food safety concern scale positively affects attitude toward using the scale.

*H6*: The food safety concern scale positively affects the subjective norm scale.

### Theory of reasoned action

The TRA was developed by Fishbein and Ajzen (28), arguing that will and intention predict behavior. The TRA ‘traces causal links from beliefs to attitudes and intentions to actual behavior’ and is used to explain ‘behavior that is largely under voluntary control’ (90). The TRA suggests that people have higher intentions (motivation) and are more likely to act on the proposed behavior if they have a good attitude toward the behavior and believe that others want them to undertake the behavior (subjective norm). Therefore, ‘it is not necessary that positive attitudes toward behavior result in actual behavior unless there is group persuasion or coercion from one’s immediate social environment or vice versa’ (58). Several studies investigating consumers’ food consumption intentions and behavior have shown the TRA to be a theoretical basis (22, 32–34).

### Theory of planned behavior

Widely used and developed in social psychology, TPB is the theory developed by Ajzen (35) based on the TRA previously developed by Fishbein and Ajzen (28). TPB adds a component that can consider both actual and perceived challenges that a person may experience regarding the act of performing (or not performing) a particular behavior, even though both theories require that people’s behavior is based on deliberative grounds (e.g., consideration of the consequences of a particular action) (49). TPB assumes that behaviors are influenced by intentions determined by attitudes, SNs, and perceived behavioral control (35). Furthermore, the relative significance of each factor in predicting an individual’s conduct varies between actions and circumstances (57). Like TRA, TPB assumes that a particular behavior is determined by the intention to perform it (51). Many studies investigating consumers’ food consumption intentions and behaviors have shown TPB as a theoretical basis (22, 26, 49, 52).

### Attitude toward using

Attitude refers to a psychological disposition that defines an individual’s self-performance evaluation and predicts intentions and actual behavior (28). Since attitudes have been demonstrated to significantly influence and predict an extensive variety of behavior, they are a crucial psychological concept. Attitudes are relatively permanent and stable summaries of an item’s judgment (91). According to Sparks and Shepherd (92), attitudes also influence healthy and organic food purchasing behavior as this construct influences much consumer behavior. This assertion is supported by the view that attitude is the primary determinant of such purchasing behavior (33, 73). Consumer attitudes towards food safety can be differentiated according to the type of food safety issues of concern (7). At this point, Brewer and Prestat (93) found that chemical issues, spoilage issues, health issues, regulatory issues and deceptive practices influence attitudes towards food safety. Therefore, consumers’ perceptions of food safety to protect their health are in the direction of accessing healthy, environmental, and organic food (9, 94). During the pandemic period, the scope of these existing perceptions has narrowed, and green food (95) and green procurement (96) approaches have played an active role in directing consumer attitudes. There are also studies in the literature that link HC with ATU. In one of these studies, Khayyam et al. (9) found that HC positively affects consumers’ attitudes towards use. Lin and Wu (97) suggested that health-conscious consumers’ attitudes towards healthy and natural foods align with the study. The following hypotheses were developed in line with the literature research:

*H9*: The health consciousness scale positively affects the attitude scale through the food safety knowledge scale.

*H11*: The health consciousness scale positively affects the attitude scale through the FSC scale.

### Subjective norm

In addition to attitudinal influence, social influence also plays a role in specific food consumption behavior, corresponding to the SN (9). SNs represent the perceived external pressure on individuals to engage in actions or not (28, 35). Furthermore, the SN shows how individuals think that people who are important to them should behave (for example, acting to protect food safety) (30). Regarding food safety, these norms reflect individuals’ expectations in the observance of food safety and individual motivations to comply with these expectations (46). The perception of food safety also has a subjective aspect, and whether a person will act is based on the opinions of people who are important to him/her (98). Several studies suggest that SNs primarily predict food safety BI (52). SNs have been associated with the intention to purchase green and healthy products (77) and directly affect a willingness to try nutritional food supplements (99). In addition, Roberts, and Barrett (40) suggest that managers’ SNs largely shape restaurant managers’ perceptions of food safety. In line with the literature review, the following hypotheses were developed:

*H10*: The health consciousness scale positively affects the subjective norm scale through the food safety knowledge scale.

*H12*: The health consciousness scale positively affects the subjective norm scale through the food safety concern scale.

### Behavioral intention

BI is an action that a person plans or hopes to take in the future (100), and according to Fishbein and Ajzen (101), it is a probability of action that represents an individual’s expectation of a particular action in a specific setting. It has been observed that attitudes toward all behavioral alternatives, as compared to attitudes toward a single of the possible actions, can more accurately predict BIs in a choice situation (102). Intention can therefore be used as an approximate substitute for behavior when a measure of actual behavior is not easily available, even though there is some variation between BI and actual behavior (103). The prediction of food safety behavior is made possible by the implementation of the BI for food safety. Furthermore, it provides an explanation of action by considering motivational antecedents in addition to additional individual and cognitive aspects (52). One such behavior is food purchasing behavior. For example, several scholars have suggested that health concern determines consumers’ BI to consume healthy food (94, 104). Another factor affecting the BI of food consumption is food safety. At this point, Lin and Wu (83) found that consumers’ perception of food safety in restaurants affects their BIs. Khayyam et al. (9) show that HC has an indirect effect on BI to purchase food in addition to food safety. In line with the literature research, the following hypotheses were developed:

*H7*: Attitude toward using the scale positively affects the behavioral intention scale.

*H8*: The subjective norm scale positively affects the behavioral intention scale.

*H13*: The health consciousness scale positively affects the behavioral intention scale through the sequential mediation of the food safety knowledge scale and attitudes toward using the scale.

*H14*: The health consciousness scale positively affects the behavioral intention scale with the sequential mediation of food safety knowledge scale and the subjective norm scale.

*H15*: The health consciousness scale positively affects the behavioral intention scale with the sequential mediation of food safety concern scale and attitudes toward using the scale.

*H16*: The health consciousness scale positively affects the behavioral intention scale with the sequential mediation of food safety concern scale and the subjective norm scale.

## Methodology

### Research population and sample

The study population consists of consumers in Turkey who take food supplements. The study used a convenient sampling method. Kline (105) suggests that to perform structural equation modelling, a minimum sample size of 200 is required. Therefore, the sample population involved 327 participants. Total of 327 people who could be included in the analysis, 55.4% were male and 44.6% were female. There is a distribution of 8.6% between the ages of 18–20 years, 28.7% between the ages of 31–40 years, 9.8% between the ages of 41–50 years and 1.5% between the ages of 51–60 years. According to descriptive analysis, most of the participants generally used the food supplements of Vitamins (60.6%), Minerals (42.5%), and Omega3/Fish Oils (30%).

### Collection of research data

The data for the study was collected using the online survey method. The questionnaire’s design adopted a five-point Likert-type scale (1: strongly disagree to 5: strongly agree). The research data were collected in the third quarter of 2023. In the first part of the research questionnaire, several pre-test questions regarding the intake of food supplements were submitted to determine the convenience of the participants. In the second part, questions were asked regarding the variables of Health Awareness, Food Safety Knowledge, Food Safety Concern, and Theory of Reasoned Action (Attitude, Subjective Norm, and Behavioral Intention). In the third part, some questions were asked regarding age, gender, education, and income status to obtain information about the demographic variables of the participants.

### Conceptual model and scale development

**Figure 1:**
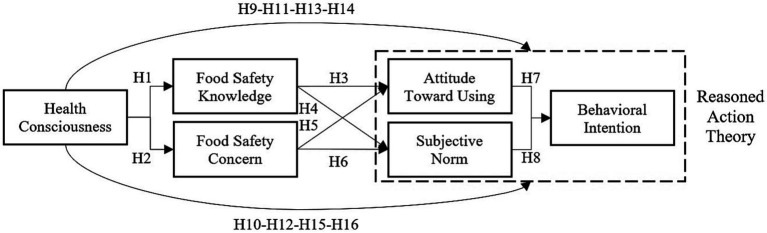
Conceptual model.

The conceptual model is included six items. The HC scale is adapted from Nagaraj (63) and consists of 5 statements. The FSK scale is adapted from Latip et al. (80) and consists of 3 statements. The FSC scale is adapted from Bhutto et al. (73) and consists of 4 statements. The TRA (ATU, SN, BI) scale is adapted from Sen et al. (106) and Lim and An (98) and consists of 10 statements. The designed conceptual model is shown in Figure 1.

### Analysis of the research data

The primary purpose of the research is to determine how consumers’ HC levels affect their attitudes and SNs toward food supplements. The second purpose is to measure the mediating effect of FSCs and FSK. The data analysis process was completed with the help of SPSS 23, MS Excel, and Smart-PLS software to test the theoretical framework. Various statistical techniques were used to verify the validity and reliability of the questionnaire. In this direction, combined reliability (CR), Cronbach’s alpha (Cα), and average explained variance (AVE) were examined. The relationships between hypothesized items in the structural model were tested using the Smart-PLS. Confirmatory factor analysis (CFA) was used to evaluate the measurement model. The regression analyses were performed in the structural model phase to evaluate overall fit and suggested relationships by way of Smart-PLS.

## Results

### Measurement model results

CFA was used to demonstrate the accuracy and validity of the model used in this study. Since the scales’ distributions were unsuitable for normal distribution (*p* < 0.05), the analyses were performed using Smart-PLS software, which analyses non-parametric assumptions.

As a result of the CFA, model fit measures were calculated as Ki Kare (*χ*^2^)/*df* = 2.451, Incremental Fit Index (IFI) = 0.935, Normed Fit Index (NFI) = 0.894, CFI = 0.934, Goodness of Fit Index (GFI) = 0.881, Adjustment Goodness of Fit Index (AGFI) = 0.845, Turker-Lewis Index (TLI) = 0.921 and Root Mean Square Error of Approximation (RMSEA) = 0.067. According to the model fit criteria, the NFI, GFI and AGFI statistics are below the threshold value (0.900). It has been calculated that these statistics tend to fall to 0.80 levels and below (107) in cases where the sample size is <500 or according to a change in the number of scales (108). According to this information, the model was considered suitable for evaluation in its current form (109).

The calculations for the internal consistency of the scales are presented in Table 1. The fact that the loadings of the scales according to the statements of the scales were calculated above 0.6, the AVE statistic was between 0.670–0.817, and the CR statistic was between 0.859–0.932, shows that the internal consistency and fit of the scales are sufficient (110). Based on this, it is interpreted that factorization is appropriate, and the existing statements can be combined with their scales.

**Table 1:** Cronbach alpha, composite reliability, average variance extracted.

Constructs	Item loading	Mean	SD	Cronbach’s alpha	CR	AVE
**Health consciousness (HC)**		**3.99**	**0.72**	**0.895**	**0.923**	**0.704**
I’m very self-conscious about my health	0.822					
I’m usually aware of my health	0.851					
I’m aware of the state of my health as I go through the day	0.837					
I’m alert to changes in my health	0.822					
I take responsibility for the state of my health	0.863					
**Food safety concern (FSC)**		**4.19**	**0.77**	**0.763**	**0.865**	**0.682**
Nowadays most foods contain residues from chemical sprays and fertilizers.	0.749					
I am very concerned about the number of preservatives in food	0.904					
The quality and safety of food nowadays concerns me.	0.816					
**Food safety knowledge (FSK)**		**3.37**	**0.92**	**0.902**	**0.932**	**0.773**
Knowledgeable about food safety	0.884					
Knowledgeable on food safety certificates	0.881					
Knowledgeable on food selection to reduce potential foodborne illness	0.866					
Good food safety knowledge	0.886					
**Attitude toward using (ATU)**		**3.3**	**0.8**	**0.847**	**0.897**	**0.687**
Taking food supplements is healthy	0.887					
Taking food supplements is safe	0.904					
Taking food supplements is nutritious	0.740					
I buy food supplements for my family to get nutrition	0.774					
**Subjective norm (SN)**		**3.29**	**0.81**	**0.762**	**0.859**	**0.670**
My acquaintances understand me choosing food supplements as a wellbeing health	0.842					
My acquaintances think that I should take food supplements	0.791					
My acquaintances approve me taking food supplements	0.822					
**Behavioral intention (BI)**		**3.19**	**0.93**	**0.887**	**0.930**	**0.817**
I will make an effort to purchase more food supplements	0.846					
I intend to purchase food supplements in the future	0.939					
I want to purchase food supplements in the future	0.924					

Bold text shows the scale statistics.

Another step in assessing the appropriateness of the model is to demonstrate the existence of decomposition across the scales. For this purpose, the Fornell Larcker (110) and Heterotrait-Monotraits (HTMT) statistics in Table 2 are calculated. In the Fornell Larcker (110) calculation, no value may be greater than the diagonal value. In the HTMT calculation, since all values are <0.90, it is understood that the scales’ separation can be appropriately evaluated.

**Table 2:** Discriminant validity^*^ of construct.

Constructs	HC	FSC	FSK	ATU	SN	BI
HC	**0.839**					
FSC	0.424 (0.511)	**0.826**				
FSK	0.478 (0.537)	0.353 (0.432)	**0.879**			
ATU	0.218 (0.237)	0.095 (0.122)	0.247 (0.274)	**0.829**		
SN	0.221 (0.225)	0.187 (0.238)	0.303 (0.339)	0.535 (0.644)	**0.819**	
BI	0.167 (0.185)	0.104 (0.125)	0.262 (0.29)	0.672 (0.761)	0.483 (0.573)	**0.904**

^*^Fornell Larcker (HTMT); HC, health consciousness; FSC, food safety concern; FSK, food safety knowledge; SN, subjective norm; ATU, attitude toward using; BI, behavioral intention. Bold text shows the scale Fornell Larcker statistics.

### Structural modelling

**Figure 2:**
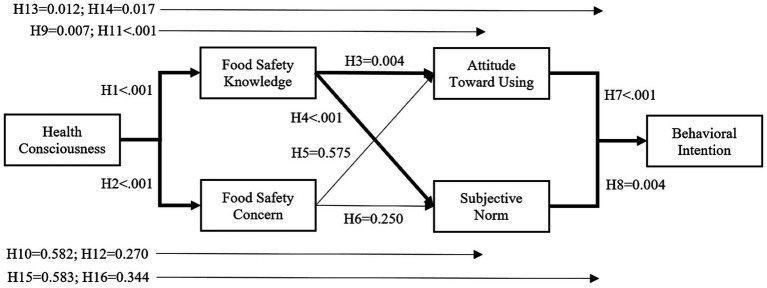
Direct effects results of structural model.

At the analysis stage, the model was tested using the bootstrapping technique. As a result of the analysis, model fit measures were calculated to be *χ*^2^/*df* = 2.777, IFI = 0.917, NFI = 0.876, CFI = 0.916, GFI = 0.869, AGFI = 0.835, TLI = 0.904 and RMSEA = 0.074. As a result of the model, the significance results of the interactions between the scales were evaluated (Figure 2). The results obtained are presented as direct effects in line with the scales in the model (Table 3) and indirect effects obtained in line with the study’s objective (Table 4). Due to the structure of the study model and the scales it includes, many significant or insignificant interactions were found. While the results directly related to the study are shown under the ‘main’ heading in Table 4, other interactions between the scales are displayed under the ‘side’, leading to increased awareness regarding the scales.

**Table 3:** Direct effects on structural model result.

Hypothesized PATHs	Coefficients	*p*	Significance (*p* < 0.05)
*H1*: HC→FSK	0.484	<0.001	Supported
*H2*: HC→FSC	0.429	<0.001	Supported
^*^HC→ATU	0.135	0.036	Supported
^*^HC→SN	0.072	0.253	Not Supported
^*^HC→BI	−0.036	0.469	Not Supported
*H3*: FSK→ATU	0.193	0.004	Supported
*H4*: FSK→SN	0.240	<0.001	Supported
^*^FSK→BI	0.085	0.120	Not Supported
*H5*: FSC→ATU	−0.034	0.575	Not Supported
*H6*: FSC→SN	0.070	0.250	Not Supported
^*^FSC→BI	0.008	0.867	Not Supported
*H7*: ATU→BI	0.574	<0.001	Supported
*H8*: SN→BI	0.157	0.004	Supported

HC, health consciousness; FSC, food safety concern; FSK, food safety knowledge; SN, subjective norm; ATU, attitude toward using; BI, behavioral intention; ^*^Interactions that are not directly included in the purpose of the study but are naturally calculated for model clarity.

**Table 4:** Structural model and hypothesis testing result.

Results	Hypothesized PATHs	Coefficients	*p*	Significance (*p* < 0.05)	Mediator model^a^
Main	*H13*: HC→FSK→ATU→BI	0.054	0.012	Supported	Full mediation
	*H14*: HC→FSK→SN→BI	0.018	0.017	Supported	Full mediation
	*H15*: HC→FSC→ATU→BI	−0.008	0.583	Not Supported	No effect
	*H16*: HC→FSC→SN→BI	0.005	0.344	Not Supported	No effect
Side	*H9*: HC→FSK→ATU	0.093	0.007	Supported	Complementary
	*H10*: HC→FSK→SN	0.116	<0.001	Supported	Full mediation
	*H11*: HC→FSC→ATU	−0.015	0.582	Not Supported	Direct only
	*H12*: HC→FSC→SN	0.030	0.270	Not Supported	No effect
	^*^HC→FSK→BI	0.041	0.128	Not Supported	No effect
	^*^HC→FSC→BI	0.003	0.870	Not Supported	No effect
	^*^HC→ATU→BI	0.077	0.038	Supported	Full mediation
	^*^HC→SN→BI	0.011	0.295	Not Supported	No effect
	^*^FSK→ATU→BI	0.111	0.008	Supported	Full mediation
	^*^FSK→SN→BI	0.038	0.012	Supported	Full mediation
	^*^FSC→ATU→BI	−0.020	0.576	Not Supported	No effect
	^*^FSC→SN→BI	0.011	0.321	Not Supported	No effect

^*^Interactions that are not directly included in the purpose of the study but are naturally calculated for model clarity. ^a^Interpreted according to the approach of Zhao et al. (111). HC, health consciousness; FSC, food safety concern; FSK, food safety knowledge; SN, subjective norm; ATU, attitude toward using; BI, behavioral intention.

It was deemed appropriate first to explain the direct interactions between the scales to perceive the working principle of the model. As can be seen in Table 3, statistically significant interactions can be observed from the HC and BI scales. First, it is calculated that the change in the HC scale causes a significant positive interaction between FSK (β = 0.484) and FSC (β = 0.429). While the change in the FSK scale interacted positively with the ATU (β = 0.193) and the SN (β = 0.240), these scales (β_ATU_ = −0.034, β_SN_ = 0.070) were not statistically affected by the change in the FSC scale. The BI scale was positively affected by the changes in both the ATU (β = 0.574) and the SN (β = 0.157). These interactions result from the relationships desired to be explained in the model. In addition, another interaction obtained is that the HC scale positively affects the ATU (β = 0.135).

The existing direct effects identified help to understand the indirect effects. The results of the tests showing indirect effects, which are the main focus of the study, can be examined in Table 4. The information in this table is displayed under the heading ‘main’ as the desired research results and under the heading ‘side’ as the additional results in finalising the research. Since the FSC scale does not affect the variables in the following layers, the existence of any direct or indirect effect with this variable is not considered statistically significant. Therefore, indirect effects may be established through the entire FSK scale.

The results of the PATH analysis show that the consumers’ HC scores affect the BI scores through the mediation of FSK, ATU and SN. It is predicted that consumers’ BI scores will change positively in both the indirect interaction through the ATU and the indirect interaction through the SN. Since the coefficient in the indirect effect through the ATU scale is larger, it can be said that there is a higher interaction in the measurements to be made in this way.

The additional results obtained from PATH analysis. These results are considered as side benefits of the main objective. According to result, FSK has a complementary partial mediating effect on the interaction between health awareness and ATU. FSK has a full mediating effect on the interaction between health awareness and SN. ATU has a full mediating effect on the interaction between health awareness and behavioral intention. Finally, ATU and SN has a full mediating effect on the interaction between FSK and behavioral intention.

## Discussion

Consumers make numerous decisions, either consciously or unconsciously. When these decisions are considered from a marketing perspective, measurements are made on consumers’ attitudes, perceptions and/or intentions. This study investigates whether consumers’ health awareness indirectly affects their BIs. As stated in the “Structural Modelling” section, many direct and indirect effects were calculated while measuring the interaction in the sequential layers from health awareness to BI.

The scales in the study have also been used in many studies in the literature. The extent to which geographical and demographic differences of consumers change their attitudes and behavior related to health and food is included in the literature (3, 9, 33, 36, 40, 63, 73, 76, 79). The current study calculates that FSK and FSC were positively affected by the HC of the participants. Similarly, it has been reported that HC is positively affected by consumers’ physical characteristics (74) and SNs (9, 74). The present study calculates that HC does not positively or negatively affect SNs. In another study, it was shown that HC directly affected purchase intention (73). In the present study, the indirect effect of HC on BI, not on purchase intention, is addressed, which is similar to Khayyam et al. (9). The existence of studies in which the health awareness and BI of consumers are positive (9, 60, 63, 73) and the parallel results obtained in the current study indicate that consumers’ concerns about their health have increased. Moreover, it also shows us that these concerns increase their behavior to eliminate them. It is understood that the results obtained in the present study show parallels with similar studies in the literature for the last decade.

While testing the present model, it was determined that scores related to FSK and concern could affect BIs. The results obtained in the research that inspired the FSK scale (80) are similar to the findings obtained in the current study. Accordingly, FSK positively influences ATU. FSK also positively affects the SN variable in our study. In addition, a study in Asia reported that consumers’ FSK positively influences their intention to purchase organic food (82). Similarly, Sanlier and Konaklioglu (84) reported a significant correlation between food handling and food knowledge, even if the interaction was not calculated in their study. From this point of view, the results obtained in the present study are in parallel with the reflections in the literature. As a consequence of the study, as consumers’ FSK scores increase, it is understood that the ATU and even the SN scores of the same consumers may also change positively.

It is a well-known fact that knowledge or concern regarding any subject will not affect the behavior of individuals in the same way. From this point of view, it is essential to include FSC and FSK in the study. Our study calculates that the change in FSC does not have a significant effect on the ATU or the change in SNs. The current approach contradicts several studies (9, 60, 73) that have addressed purchase intention. We speculate that this may be due to the characteristics of the participants as well as other unknown sociodemographic, economic, and other variables. In the current study, all direct effects with the FSC variable in the model were insignificant. Therefore, the indirect effects were also found to be insignificant.

Another intermediary layer of the TRA, which is the basic theory in the study, includes ATU and SNs. In this model, the direct effect of ATUs and SNs on BI is an expected phenomenon in the research design. As predicted, it was calculated that consumers’ ATU and SN scores positively affect their intention to use food supplements. The findings of the studies in the literature also coincide with the current study’s findings in this direction. In studies on the purchase of organic foods (33, 92, 97), it was reported that consumers’ attitudes towards use, and SN scores increased purchase intention. In contrast to the results, Milton and Mullan (46) argue that ATU, and SN do not affect BI; instead, perceived behavioral control does. In addition, Lin and An (98) suggest that perceived behavioral control is the most influential factor on BI to purchase Yak-Sun foods. Based on the result obtained, it is thought that personal norms may be more influenced by cultural structure due to the individual and cultural characteristics of the participants in the current study and the characteristics of the participants in similar studies in the literature (33, 46, 92, 97, 98). At the same time, the ATU is more independent of culture.

The focus of the study is to determine how consumers’ BIs to use supplements are influenced. When the variables in the current study were examined, it was concluded that BI to use supplements was directly influenced by ATU and SN. In contrast, it was indirectly influenced by HC and FSK. In the literature, there are studies in which BI to use supplements is directly affected by ATU and SN (9, 36, 59, 60, 80, 102) and indirectly affected by FSK (9, 60, 78, 94). Therefore, it is understood that the current study’s behavioral influences align with the literature. Based on this, consumers have a positive attitude towards supplement use. It can be interpreted that having information about the foods consumed and having concerns about personal health leads consumers to use supplements. Therefore, it is thought that supplement use will increase in the future (even if it is not the subject of the study). Regarding marketing discipline, it would be a reasonable inference to interpret that the commercial volume and expected market size of supplements will also increase.

## Conclusion

Consumers prefer food supplements for several reasons, especially in recent years. This research attempts to explain the underlying reasons for consumers’ use in terms of certain variables. The relevant literature has been researched theoretically and empirically, and several hypotheses have been created. The primary purpose of the research is to determine how consumers’ HC levels affect their ATUs and SNs toward food supplements. The second purpose is to measure the mediating effect of FSCs and FSK.

In light of the results, we can say that the HC variable affects the FSK and FSC variables significantly and positively. The FSK variable affects the attitude toward using SN variables significantly and positively. ATU and SN variables affect the BI variable significantly and positively. Finally, it was found that the relationship between HC and ATU/SN variables was mediated by the FSK variable. In other words, it affects the attitudes and behavior of consumers with high HC regarding the use of food supplements through the mediating effect of FSK.

Consequently, the state’s actions on food supplement inspection are crucial since terms such as HC and food supplement safety directly affect public health. This research provides findings that have a multiplier effect on health institutions and organizations to publish a digital health declaration/report on food supplements, prepare a sustainable consumption model, or direct them to organic food supplements. In this respect, it is thought that the relevant research will be an essential guide for academicians, researchers, and practitioners.

### Limitations and future research

The sample of the research is limited to 327 participants. These participants were selected from people living in Turkey and who use food supplements. In future research, more comprehensive analyses could be conducted on people living in different countries and cultures. Additionally, certain age, gender, and intergenerational comparisons could be made. The survey method, which is a quantitative method, was used to collect research data. In subsequent research, qualitative methods, such as ethnography, focus group interviews, and observations, may also be preferred. The Smart PLS package program was used to analyse the research data. The research model was designed based on the TRA. The TPB, a more developed version of the TRA, could also be used in future research. In this regard, consumers’ reactions to the Perceived Behavior Control variable may also be measured. The product group that the research focuses on is food supplements. Subsequent research could be conducted on different product and brand groups. Product groups such as dietary supplements, organic foods and vegan foods could also be given as examples. The studies can be conducted to investigate the intention of consumers with different diet types or different health problems to use food supplements. Consumers with professions that professionally involve food processing and/or food safety concepts can be examined within this scope. It is thought that studies that will focus on the perceptions and usage intentions of health professionals on food supplements will make a concrete contribution. In addition, the approaches of consumers with religious, cultural, and geographical restrictions to the use of food supplements can also be examined.

## Data availability statement

The original contributions presented in the study are included in the article/Supplementary material, further inquiries can be directed to the corresponding author.

## Ethics statement

The studies involving humans were approved by Sirnak University Ethics Committee, which is numbered 2023/68004/1 and dated 16.06.2023. The studies were conducted in accordance with the local legislation and institutional requirements. The participants provided their written informed consent to participate in this study.

## Author contributions

TB: Investigation, Methodology, Project administration, Supervision, Writing – original draft, Writing – review & editing. SÇ: Data curation, Software, Validation, Visualization, Writing – original draft, Writing – review & editing. MFT: Conceptualization, Investigation, Resources, Writing – original draft, Writing – review & editing.

## References

[1] Chinea C Suárez E Hernández B. Meaning of food in eating patterns. Br Food J. (2020) 122:3331–41. doi: 10.1108/BFJ-02-2020-0144

[2] Maxwell S Shaw J. Food, food security and un reform. IDS Bull. (1995) 26:41–53. doi: 10.1111/j.1759-5436.1995.mp26004008.x

[3] Liguori J Trübswasser U Pradeilles R Le Port A Landais E Talsma EF. How do food safety concerns affect consumer behaviors and diets in low- and middle-income countries? A systematic review. Global. Food Security. (2022) 32:100606. doi: 10.1016/j.gfs.2021.100606

[4] World Health Organization. Food Safety; (2022). Available at: https://www.who.int/news-room/fact-sheets/detail/food-safety (Accessed July 18, 2023).

[5] Regusci M. FoodSafetyTech. The Costs of Food Safety: Correction versus Prevention. (2022). Available at: https://foodsafetytech.com/column/the-costs-of-food-safety-correction-vs-prevention/ (Accessed July 30, 2023).

[6] Knight A Warland R. The relationship between sociodemographics and concern about food safety issues. J Consum Aff. (2004) 38:107–20. doi: 10.1111/j.1745-6606.2004.tb00467.x

[7] Wilcock A Pun M Khanona J Aung M. Consumer attitudes, knowledge and behaviour: a review of food safety issues. Trends Food Sci Technol. (2004) 15:56–66. doi: 10.1016/j.tifs.2003.08.004

[8] Pinstrup-Andersen P. Food security: definition and measurement. Food Sec. (2009) 1:5–7. doi: 10.1007/s12571-008-0002-y

[9] Khayyam M Chuanmin S Qasim H Ihtisham M Anjum R Jiaxin L. Food consumption behavior of Pakistani students living in China: the role of food safety and health consciousness in the wake of coronavirus disease 2019 pandemic. Front. Psychol. (2021) 12:673771. doi: 10.3389/fpsyg.2021.673771, PMID: 34385954 PMC8353093

[10] Simelane KS Worth S. Food and nutrition security theory. Food Nutr Bull. (2020) 41:367–79. doi: 10.1177/0379572120925341, PMID: 33200627

[11] Unusan N. Consumer food safety knowledge and practices in the home in Turkey. Food Control. (2007) 18:45–51. doi: 10.1016/j.foodcont.2005.08.006

[12] Kennedy J Jackson V Cowan C Blair I McDowell D Bolton D. Consumer food safety knowledge: segmentation of Irish home food preparers based on food safety knowledge and practice. Br Food J. (2005) 107:441–52. doi: 10.1108/00070700510606864

[13] Medeiros LC Hillers VN Chen G Bergmann V Kendall P Schroeder M. Design and development of food safety knowledge and attitude scales for consumer food safety education. J Am Diet Assoc. (2004) 104:1671–7. doi: 10.1016/j.jada.2004.08.030, PMID: 15499353

[14] Bruhn CM Schutz HG. Consumer food safety knowledge and practices. J Food Saf. (1999) 19:73–87. doi: 10.1111/j.1745-4565.1999.tb00235.x

[15] Nesbitt A Thomas MK Marshall B Snedeker K Meleta K Watson B. Baseline for consumer food safety knowledge and behaviour in Canada. Food Control. (2014) 38:157–73. doi: 10.1016/j.foodcont.2013.10.010

[16] Quick V Corda KW Chamberlin B Schaffner DW Byrd-Bredbenner C. Ninja kitchen to the rescue: evaluation of a food safety education game for middle school youth. Br Food J. (2013) 115:686–99. doi: 10.1108/00070701311331481

[17] Byrd-Bredbenner C Abbot JM Quick V. Food safety knowledge and beliefs of middle school children: implications for food safety educators. J Food Sci Educ. (2010) 9:19–30. doi: 10.1111/j.1541-4329.2009.00088.x

[18] Liu P Lee YM. An investigation of consumers’ perception of food safety in the restaurants. Int J Hosp Manag. (2018) 73:29–35. doi: 10.1016/j.ijhm.2018.01.018

[19] Seaman P Eves A. The management of food safety—the role of food hygiene training in the UK service sector. Int J Hosp Manag. (2006) 25:278–96. doi: 10.1016/j.ijhm.2005.04.004

[20] Nagar K. An examination of gym supplement choice: using the modified theory of planned behaviour. J Food Prod Mark. (2020) 26:499–520. doi: 10.1080/10454446.2020.1817827

[21] Liu C Sun CK Chang YC Yang SY Liu T Yang CC. The impact of the fear of COVID-19 on purchase behavior of dietary supplements: integration of the theory of planned behavior and the protection motivation theory. Sustainability. (2021) 13:12900. doi: 10.3390/su132212900

[22] Zhu Y Wen X Chu M Sun S. Consumers’ intention to participate in food safety risk communication: A model integrating protection motivation theory and the theory of reasoned action. Food Control. (2022) 138:108993. doi: 10.1016/j.foodcont.2022.108993

[23] Chen MF. Extending the protection motivation theory model to predict public safe food choice behavioural intentions in Taiwan. Food Control. (2016) 68:145–52. doi: 10.1016/j.foodcont.2016.03.041

[24] Hanson JA Benedict JA. Use of the health belief model to examine older adults’ food-handling behaviors. J Nutr Educ Behav. (2002) 34:S25–30. doi: 10.1016/S1499-4046(06)60308-4, PMID: 12047826

[25] Yan C Siddik AB Masukujjaman M Dong Q Hamayun M Guang-Wen Z. Bi-dimensional values and attitudes toward online fast food-buying intention during the COVID-19 pandemic: An application of VAB model. Front Nutr. (2022) 9:894765. doi: 10.3389/fnut.2022.894765, PMID: 36505256 PMC9733427

[26] Hamid S Azhar M Sujood. Behavioral intention to order food and beverage items using e-commerce during COVID-19: an integration of theory of planned behavior (TPB) with trust. Br Food J. (2022) 125:112–31. doi: 10.1108/BFJ-03-2021-0338

[27] Mullan BA. Knowledge, Beliefs and Attitudes, Concerning Food Hygiene, in Children and Young Adults, in South East Wales. Ann Arbor, United States: ProQuest LLC (1997).

[28] Fishbein M Ajzen I. Attitudes and voting behavior: an application of the theory of reasoned action In: Stephenson GM Davis M, editors. Progress in Applied Social Psychology. London: Wiley (1981). 253–313.

[29] Hinsz VB Nickell GS. The prediction of workers’ food safety intentions and behavior with job attitudes and the reasoned action approach. Rev Psicol Trab Organ. (2015) 31:91–100. doi: 10.1016/j.rpto.2015.03.001

[30] Hinsz VB Nickell GS Park ES. The role of work habits in the motivation of food safety behaviors. J Exp Psychol Appl. (2007) 13:105–14. doi: 10.1037/1076-898X.13.2.105, PMID: 17535135

[31] Silk KJ Weiner J Parrott RL. Gene cuisine or Frankenfood? The theory of reasoned action as an audience segmentation strategy for messages about genetically modified foods. J Health Commun. (2005) 10:751–67. doi: 10.1080/10810730500326740 16316937

[32] Hussain I Rahman SU Zaheer A Saleem S. Integrating factors influencing consumers’ halal products purchase: application of theory of reasoned action. J Int Food Agribus Mark. (2016) 28:35–58. doi: 10.1080/08974438.2015.1006973

[33] Agarwal P. Theory of reasoned action and organic food buying in India. Srusti Manag Rev. (2019) 11:28–37.

[34] Gundala RR Nawaz NRMH Boobalan K Gajenderan VK. Does gender moderate the purchase intention of organic foods? Theory of reasoned action. Heliyon. (2022) 8:e10478. doi: 10.1016/j.heliyon.2022.e10478, PMID: 36097479 PMC9463379

[35] Ajzen I. The theory of planned behavior. Organ Behav Hum Decis Process. (1991) 50:179–211. doi: 10.1016/0749-5978(91)90020-T

[36] Rezaei R Mianaji S Ganjloo A. Factors affecting farmers’ intention to engage in on-farm food safety practices in Iran: extending the theory of planned behavior. J Rural Stud. (2018) 60:152–66. doi: 10.1016/j.jrurstud.2018.04.005

[37] Soon JM Baines RN. Food safety training and evaluation of handwashing intention among fresh produce farm workers. Food Control. (2012) 23:437–48. doi: 10.1016/j.foodcont.2011.08.012

[38] York VK Brannon LA Roberts KR Shanklin CW Howells AD. Using the theory of planned behavior to elicit restaurant employee beliefs about food safety: using surveys versus focus groups. J Foodserv Bus Res. (2009) 12:180–97. doi: 10.1080/15378020902910777

[39] Pilling VK Brannon LA Shanklin CW Howells AD Roberts KR. Identifying specific beliefs to target to improve restaurant employees’ intentions for performing three important food safety behaviors. J Am Diet Assoc. (2008) 108:991–7. doi: 10.1016/j.jada.2008.03.014, PMID: 18502232

[40] Roberts KR Barrett BB. Restaurant managers’ beliefs about food safety training: An application of the theory of planned behavior. J Foodserv Bus Res. (2011) 14:206–25. doi: 10.1080/15378020.2011.594379

[41] Lobb AE Mazzocchi M Traill WB. Modelling risk perception and trust in food safety information within the theory of planned behaviour. Food Qual Prefer. (2007) 18:384–95. doi: 10.1016/j.foodqual.2006.04.004

[42] Mullan BA Wong CL. Hygienic food handling behaviours. An application of the theory of planned behaviour. Appetite. (2009) 52:757–61. doi: 10.1016/j.appet.2009.01.007 19501776

[43] Mullan BA Wong C Kothe EJ. Predicting adolescents’ safe food handling using an extended theory of planned behavior. Food Control. (2013) 31:454–60. doi: 10.1016/j.foodcont.2012.10.027

[44] Mucinhato RMD da Cunha DT Barros SCF Zanin LM Auad LI Weis GCC. Behavioral predictors of household food-safety practices during the COVID-19 pandemic: extending the theory of planned behavior. Food Control. (2022) 134:108719. doi: 10.1016/j.foodcont.2021.108719, PMID: 34961805 PMC8695225

[45] Archila-Godínez JC Chen H Klinestiver L Rosa L Barrett T Henley SC. An evaluation of a virtual food safety program for low-income families: applying the theory of planned behavior. Foods. (2022) 11:355. doi: 10.3390/foods11030355 35159504 PMC8834591

[46] Milton AC Mullan BA. An application of the theory of planned behavior—a randomized controlled food safety pilot intervention for young adults. Health Psychol. (2012) 31:250–9. doi: 10.1037/a0025852, PMID: 22059618

[47] Riebl SK Estabrooks PA Dunsmore JC Savla J Frisard MI Dietrich AM. A systematic literature review and meta-analysis: the theory of planned Behavior’s application to understand and predict nutrition-related behaviors in youth. Eat Behav. (2015) 18:160–78. doi: 10.1016/j.eatbeh.2015.05.016, PMID: 26112228

[48] Blanke J Billieux J Vögele C. Healthy and sustainable food shopping: a survey of intentions and motivations. Front Nutr. (2022) 9:742614. doi: 10.3389/fnut.2022.742614, PMID: 35308289 PMC8924458

[49] Scalco A Noventa S Sartori R Ceschi A. Predicting organic food consumption: a meta-analytic structural equation model based on the theory of planned behavior. Appetite. (2017) 112:235–48. doi: 10.1016/j.appet.2017.02.007, PMID: 28188865

[50] Al-Swidi A Mohammed Rafiul Huque S Haroon Hafeez M Noor Mohd Shariff M. The role of subjective norms in theory of planned behavior in the context of organic food consumption. Br Food J. (2014) 116:1561–80. doi: 10.1108/BFJ-05-2013-0105

[51] Canova L Bobbio A Manganelli AM. Buying organic food products: the role of trust in the theory of planned behavior. Fron Psychol. (2020) 11:575820. doi: 10.3389/fpsyg.2020.575820, PMID: 33192881 PMC7644777

[52] Lin N Roberts KR. Using the theory of planned behavior to predict food safety behavioral intention: a systematic review and meta-analysis. Int J Hosp Manag. (2020) 90:102612. doi: 10.1016/j.ijhm.2020.102612

[53] Shah Alam S Mohamed SN. Applying the theory of planned behavior (TPB) in halal food purchasing. Int J Commer Manag. (2011) 21:8–20. doi: 10.1108/10569211111111676

[54] Uygurtürk H Candan SS. Planlı Davranış Teorisi Kapsamında Tüketicilerin Besin Destek Ürünlerini Satın Alma Eğilimlerinin İncelenmesi. Üçüncü Sektör Sosyal Ekonomi. (2023) 58:72–96. doi: 10.15659/3.sektor-sosyal-ekonomi.23.01.2033

[55] Fernández-Segovia I Pérez-Llácer A Peidro B Fuentes A. Implementation of a food safety management system according to ISO 22000 in the food supplement industry: A case study. Food Control. (2014) 43:28–34. doi: 10.1016/j.foodcont.2014.02.042

[56] Bensa M Vovk I Glavnik V. Resveratrol food supplement products and the challenges of accurate label information to ensure food safety for consumers. Nutrients. (2023) 15:474. doi: 10.3390/nu15020474, PMID: 36678345 PMC9861762

[57] Tajeddini K Mostafa Rasoolimanesh S Chathurika Gamage T Martin E. Exploring the visitors’ decision-making process for Airbnb and hotel accommodations using value-attitude-behavior and theory of planned behavior. Int J Hosp Manag. (2021) 96:102950. doi: 10.1016/j.ijhm.2021.102950

[58] Yadav SS Kar SK Rai PK. Why do consumers buy recycled shoes? An amalgamation of the theory of reasoned action and the theory of planned behaviour. Front Environ Sci. (2022) 10:1007959. doi: 10.3389/fenvs.2022.1007959

[59] Michaelidou N Hassan LM. The role of health consciousness, food safety concern and ethical identity on attitudes and intentions towards organic food. Int J Consum Stud. (2008) 32:163–70. doi: 10.1111/j.1470-6431.2007.00619.x

[60] Hsu SY Chang CC Lin TT. An analysis of purchase intentions toward organic food on health consciousness and food safety with/under structural equation modeling. Br Food J. (2016) 118:200–16. doi: 10.1108/BFJ-11-2014-0376

[61] Quick V Corda K Byrd-Bredbenner C. Food safety knowledge, attitudes, behaviors and intended behaviors of middle schoolers. FASEB J. (2012) 26:814.8–8.

[62] Su Y Khaskheli A Raza SA Yousufi SQ. How health consciousness and social consciousness affect young consumers purchase intention towards organic foods. Manag Environ Q Int J. (2022) 33:1249–70. doi: 10.1108/MEQ-12-2021-0279

[63] Nagaraj S. Role of consumer health consciousness, food safety & attitude on organic food purchase in emerging market: A serial mediation model. J Retail Consum Serv. (2021) 59:102423. doi: 10.1016/j.jretconser.2020.102423

[64] Shafieizadeh K Alotaibi S Tao CW. Information processing of food safety messages: what really matters for restaurant customers? Int J Contemp Hosp Manag. (2023) 35:3638–61. doi: 10.1108/IJCHM-05-2022-0670

[65] Нургазы Ш Сейтказиева А Симанавичене Ж Рахматуллаева Д. How food security influence well-being: a moderated mediation model. Казахстан Спектр. (2022) 101:100–10. doi: 10.52536/2415-8216.2022-1.07

[66] Schutz H Read M Bendel R Bhalla V Harrill I Monagle J. Food supplement usage in seven Western states. Am J Clin Nutr. (1982) 36:897–901. doi: 10.1093/ajcn/36.5.897, PMID: 7137073

[67] European Food Safety Authority (EFSA). Food Supplements; (2022). Available at: https://www.efsa.europa.eu/en/topics/topic/food-supplements (Accessed July 18, 2023).

[68] Willis E Royne SM. Health consciousness or familiarity with supplement advertising: what drives attitudes toward dietary supplements? Int J Pharm Healthc Mark. (2016) 10:130–47. doi: 10.1108/IJPHM-06-2015-0026

[69] Cencic A Chingwaru W. The role of functional foods, nutraceuticals, and food supplements in intestinal health. Nutrients. (2010) 2:611–25. doi: 10.3390/nu2060611, PMID: 22254045 PMC3257668

[70] Stoś K Woźniak A Rychlik E Ziółkowska I Głowala A Ołtarzewski M. Assessment of food supplement consumption in polish population of adults. Front Nutr. (2021) 8:733951. doi: 10.3389/fnut.2021.733951, PMID: 34778335 PMC8578692

[71] Grand View Research. Dietary Supplements Market Size and Trends Report; (2023). Available at: https://www.grandviewresearch.com/industry-analysis/dietary-supplements-market (Accessed August 19, 2023).

[72] Becker MH Maiman LA Kirscht JP Haefner DP Drachman RH. The health belief model and prediction of dietary compliance: A field experiment. J Health Soc Behav. (1977) 18:348–66. doi: 10.2307/2955344, PMID: 617639

[73] Bhutto MY Zeng F Khan MA Ali W. Chinese consumers’ purchase intention for organic meat: An extension of the theory of planned behaviour. Asian Aca Manag J. (2022) 27:153–73. doi: 10.21315/aamj2022.27.1.7

[74] Buhrau D Ozturk TC. Motivating healthy eating: the role of presentation format and health consciousness. Food Qual Prefer. (2018) 64:167–71. doi: 10.1016/j.foodqual.2017.09.011

[75] Landström E Hursti UKK Becker W Magnusson M. Use of functional foods among Swedish consumers is related to health-consciousness and perceived effect. Br J Nutr. (2007) 98:1058–69. doi: 10.1017/S0007114507761780, PMID: 17640416

[76] DiPietro RB Remar D Parsa HG. Health consciousness, menu information, and consumers’ purchase intentions: An empirical investigation. J Foodserv Bus Res. (2016) 19:497–513. doi: 10.1080/15378020.2016.1189744

[77] Teng P Rezai G Mohamed Z Shamsudin M. Consumers’ Intention to Purchase Green Foods in Malaysia. (2011). Available at: https://www.semanticscholar.org/paper/Consumers'-Intention-to-Purchase-Green-Foods-in-Teng-Rezai/95886e53d877726a296c4c93f4e1310ee721c65c (Accessed August 20, 2023).

[78] Latip MSA Newaz FT Ramasamy R Tumin SA Noh I. How do food safety knowledge and trust affect Individual’s green considerations during the COVID-19 pandemic in Malaysia? Malasian J Consum Fam Econ. (2020) 24:261–85.

[79] Ajayi OA Salaudeen T. Consumer food safety awareness and knowledge in Nigeria. Agric J. (2014) 9:191–8.

[80] Latip MSA Newaz FT Mohamad MA Tumin SA Rahman NFA Noh I. The moderating effect of food safety knowledge on organic food purchase intention in a new normal. Pertanika J Soc Sci Hum. (2021) 29:2281–99.

[81] Adawiyah R Najib M Ali MM. Information effect on organic vegetable purchase interest through consumer preferences and awareness. J Asian Fin Econ Bus. (2021) 8:1055–62. doi: 10.13106/jafeb.2021.vol8.no2.1055

[82] Kashif U Hong C Naseem S Khan WA Akram MW. Consumer preferences toward organic food and the moderating role of knowledge: a case of Pakistan and Malaysia. Cienc Rural. (2020) 50:e20190842. doi: 10.1590/0103-8478cr20190842

[83] Chan SW Ismail F Ahmad MF Ramlan R Ng LT. Assessing food safety and food hygiene practices among tertiary students. AIP Conf Proc. (2022) 2644:030042. doi: 10.1063/5.0104751

[84] Sanlier N Konaklioglu E. Food safety knowledge, attitude and food handling practices of students. Br Food J. (2012) 114:469–80. doi: 10.1108/00070701211219504

[85] Yee WMS Yeung RMW Morris J. Food safety: building consumer trust in livestock farmers for potential purchase behaviour. Br Food J. (2005) 107:841–54. doi: 10.1108/00070700510629788

[86] EFSA – European Food Safety Authority. Special Eurobarometer Wave EB97.2 2022. Food Safety in the EU; (2023). Available at: https://www.efsa.europa.eu/sites/default/files/2022-09/EB97.2-food-safety-in-the-EU_report.pdf (Accessed August 25, 2023).

[87] EFSA (European Food Safety Authority). EFSA statement on the review of the risks related to the exposure to the food additive titanium dioxide (E 171) performed by the French Agency for Food, environmental and occupational health and safety (ANSES). EFSA J. (2019) 17:e05714. doi: 10.2903/j.efsa.2019.5714, PMID: 32626336 PMC7009203

[88] Sax JK. Dietary supplements are not all safe and not all food: how the low cost of dietary supplements preys on the consumer. Am J Law Med. (2015) 41:374–94. doi: 10.1177/0098858815591523, PMID: 26591824

[89] Wheatley VM Spink J. Defining the public health threat of dietary supplement fraud. Compr Rev Food Sci Food Saf. (2013) 12:599–613. doi: 10.1111/1541-4337.12033, PMID: 33412717

[90] Ajzen I. From intentions to actions: a theory of planned behavior In: Kuhl J Beckmann J, editors. Action Control: From Cognition to Behavior. Berlin, Heidelberg: Springer (1985). 11–39.

[91] Kraus SJ. Attitudes and the prediction of behavior: a meta-analysis of the empirical literature. Personal Soc Psychol Bull. (1995) 21:58–75. doi: 10.1177/0146167295211007

[92] Sparks P Shepherd R. Self-identity and the theory of planned behavior: assesing the role of identification with “green consumerism”. Soc Psychol Q. (1992) 55:388–99. doi: 10.2307/2786955

[93] Brewer MS Prestat CJ. Consumer attitudes toward food safety issues. J Food Saf. (2002) 22:67–83. doi: 10.1111/j.1745-4565.2002.tb00331.x

[94] Singh A Verma P. Factors influencing Indian consumers’ actual buying behaviour towards organic food products. J Clean Prod. (2017) 167:473–83. doi: 10.1016/j.jclepro.2017.08.106

[95] Qi X Yu H Ploeger A. Exploring influential factors including COVID-19 on green food purchase intentions and the intention–behaviour gap: a qualitative study among consumers in a Chinese context. Int J Environ Res Public Health. (2020) 17:7106. doi: 10.3390/ijerph17197106, PMID: 32998292 PMC7579444

[96] Lacombe A Quintela I Liao Y Wu VCH. Food safety lessons learned from the COVID-19 pandemic. J Food Saf. (2021) 41:e12878. doi: 10.1111/jfs.12878, PMID: 33612893 PMC7883256

[97] Lin WL Wu CC. The concerns about choice attributes and behavior intentions of consumers toward food safety restaurant. Int Bus Res. (2016) 9:11. doi: 10.5539/ibr.v9n4p11

[98] Lim HR An S. Intention to purchase well-being food among Korean consumers: An application of the theory of planned behavior. Food Qual Prefer. (2021) 88:104101. doi: 10.1016/j.foodqual.2020.104101, PMID: 33071469 PMC7553994

[99] O’Connor EL White KM. Willingness to trial functional foods and vitamin supplements: the role of attitudes, subjective norms, and dread of risks. Food Qual Prefer. (2010) 21:75–81. doi: 10.1016/j.foodqual.2009.08.004

[100] Swan JE Trawick IF. Disconfirmation of expectations and satisfaction with a retail service. J Retail. (1981) 57:49–67.

[101] Fishbein M Ajzen I. Belief, Attitude, Intention, and Behavior: An Introduction to Theory and Research. Massachusetts: Addison-Wesley (1975).

[102] Ajzen I Fishbein M. The prediction of behavioral intentions in a choice situation. J Exp Soc Psychol. (1969) 5:400–16. doi: 10.1016/0022-1031(69)90033-X

[103] Lu J Liu C Wang D Zhang X. Influence of work values on the prescribing behavioral intentions regarding antibiotic use among primary physicians in Hubei, China. Front Public Health. (2022) 10, 10:830181. doi: 10.3389/fpubh.2022.830181, PMID: 35646752 PMC9136237

[104] Ismael D Ploeger A. The potential influence of organic food consumption and intention-behavior gap on consumers’ subjective well-being. Foods Mayıs. (2020) 9:650. doi: 10.3390/foods9050650, PMID: 32443595 PMC7278807

[105] Kline RB. Principles and Practice of Structural Equation Modeling. New York, NY: Guilford Publications (2015).

[106] Şen İ Rad F Şen Ağilkaya G. Analyzing fish consumption behavior of Turkish consumers with theory of planned behavior. Thalassas. (2022) 38:929–37. doi: 10.1007/s41208-022-00435-3

[107] Cook AJ Brawer PA Vowles KE. The fear-avoidance model of chronic pain: validation and age analysis using structural equation modeling. Int Assoc Study Pain. (2006) 121:195–206. doi: 10.1016/j.pain.2005.11.018 16495008

[108] Wang K Xu Y Wang C Tan M Chen P. A corrected goodness-of-fit index (CGFI) for model evaluation in structural equation modeling. Struct Equ Model Multidiscip J. (2019) 27:735–49. doi: 10.1080/10705511.2019.1695213

[109] Byrne BM. Structural Equation Modeling with AMOS Basic Concepts, Applications, and Programming (Multivariate Applications Series). New York: Routledge (2010).

[110] Fornell C Larcker DF. Evaluating structural equation models with unobservable variables and measurement error. J Mark Res. (1981) 18:39–50. doi: 10.1177/002224378101800104

[111] Zhao X Lynch JG Jr Chen Q. Reconsidering baron and Kenny: myths and truths about mediation analysis. J Consum Res. (2010) 37:197–206. doi: 10.1086/651257

